# Postmeal Optogenetic Inhibition of Dorsal or Ventral Hippocampal Pyramidal Neurons Increases Future Intake

**DOI:** 10.1523/ENEURO.0457-18.2018

**Published:** 2019-01-28

**Authors:** Reilly Hannapel, Janavi Ramesh, Amy Ross, Ryan T. LaLumiere, Aaron G. Roseberry, Marise B. Parent

**Affiliations:** 1 Neuroscience Institute, Georgia State University, Atlanta, GA 30303; 2 Department of Biology, Georgia State University, Atlanta, GA 30303; 3 Department of Psychology, Georgia State University, Atlanta, GA 30303; 4 Department of Psychological and Brain Sciences and Iowa Neuroscience Institute, University of Iowa, Iowa City, IA 52242

**Keywords:** feeding, hippocampus, memory, postprandial, saccharin, sucrose

## Abstract

Memory of a recently eaten meal can serve as a powerful mechanism for controlling future eating behavior because it provides a record of intake that likely outlasts most physiological signals generated by the meal. In support, impairing the encoding of a meal in humans increases the amount ingested at the next eating episode. However, the brain regions that mediate the inhibitory effects of memory on future intake are unknown. In the present study, we tested the hypothesis that dorsal hippocampal (dHC) and ventral hippocampal (vHC) glutamatergic pyramidal neurons play a critical role in the inhibition of energy intake during the postprandial period by optogenetically inhibiting these neurons at specific times relative to a meal. Male Sprague Dawley rats were given viral vectors containing CaMKIIα-eArchT3.0-eYFP or CaMKIIα-GFP and fiber optic probes into dHC of one hemisphere and vHC of the other. Compared to intake on a day in which illumination was not given, inhibition of dHC or vHC glutamatergic neurons after the end of a chow, sucrose, or saccharin meal accelerated the onset of the next meal and increased the amount consumed during that next meal when the neurons were no longer inhibited. Inhibition given during a meal did not affect the amount consumed during that meal or the next one but did hasten meal initiation. These data show that dHC and vHC glutamatergic neuronal activity during the postprandial period is critical for limiting subsequent ingestion and suggest that these neurons inhibit future intake by consolidating the memory of the preceding meal.

## Significance Statement

Memory of a recently eaten meal provides a lasting record of recent intake and limits subsequent ingestion; however, the neural mechanisms underlying these mnemonic effects remain unknown. Here, we show that optogenetic inhibition of dorsal hippocampal (dHC) or ventral hippocampal (vHC) pyramidal neurons induced after the end of a sucrose, chow or saccharin meal, when the memory of the meal would be undergoing consolidation, accelerated the initiation of the next meal and, importantly, increased the amount consumed during that next meal when the neurons were no longer inhibited. These findings show that principal hippocampal neurons limit future intake and suggest that they do so by consolidating the memory of the preceding meal.

## Introduction

To date, the effort to understand the neural control of food intake has focused primarily on homeostatic and hedonic processes. In contrast, fewer studies have examined brain regions traditionally associated with other functions, such as memory. Yet, the memory of a recently eaten meal can serve as a powerful mechanism for controlling future eating behavior because it provides a record of recent intake that likely outlasts most physiologic signals generated by the eating bout. Indeed, evidence from studies with humans suggests that impairing the episodic memory of a meal increases intake at the next eating episode and that enhancing meal-related memory has the opposite effect (Robinson et al., 2013b; Higgs, 2016). Moreover, patients with episodic memory-type amnesia do not remember eating and will eat an additional meal when presented with food despite just having eaten to satiety (Hebben et al., 1985; Rozin et al., 1998; Higgs et al., 2008), and episodic memory deficits are associated with uncontrolled eating (Martin et al., 2017) and elevated body mass (Cheke et al., 2016).

The brain regions that mediate the inhibitory effects of ingestion-related memory on future intake are largely unknown. The principal cells of the hippocampus, pyramidal glutamatergic neurons, play a central role in memory (Izquierdo and Medina, 1997; Morris, 2013; Zhu et al., 2014). In particular, dorsal hippocampus (dHC) is critical for episodic memories of personal experiences (Shapiro et al., 2006; Fanselow and Dong, 2010; Barbosa et al., 2012) and ventral hippocampal (vHC) is important for emotional memory (Bannerman et al., 2004; Barkus et al., 2010; Fanselow and Dong, 2010). As ingestion-related memories contain both mnemonic components, it seems likely that both regions contribute to the memory of a meal. Moreover, both dHC and vHC neurons are anatomically positioned to form a memory of a meal because they express high concentrations of receptors for virtually every food-related signal (Lathe, 2001; Kanoski and Grill, 2015), receive neural impulses regarding energy status (e.g., taste, stomach distention; Xu et al., 2008, 2014), and project to most brain areas critical for energy regulation (Risold and Swanson, 1996; Kishi et al., 2000; Cenquizca and Swanson, 2006; Xu et al., 2014; Hsu et al., 2015b).

In support of a role for these regions in regulating future intake, we reported previously that dHC or vHC infusions of the GABA_A_ agonist muscimol given after the end of a first sucrose meal accelerated the onset of the second sucrose meal and increased the amount of sucrose consumed during that second meal (Henderson et al., 2013; Hannapel et al., 2017). The effects of muscimol are temporally imprecise (Martin, 1991; Arikan et al., 2002), and the postmeal inactivation likely persisted throughout the postprandial period, during intake of the next meal, and beyond that. As a result, it is impossible to know whether these postmeal manipulations increased the amount consumed during the next meal by disrupting memory-based processes during the postprandial period or via a non-mnemonic effect on intake during consumption of the second meal. Moreover, our prior work focused exclusively on scheduled sucrose meals presented during the light cycle. As a consequence, it is unclear whether the results extend to homeostatic eating behavior in free-feeding animals during the dark cycle and whether the findings depend on the caloric value of the food. To address these issues and to investigate the specific role of principal hippocampal pyramidal neurons, the current study used an activity-guided optogenetic approach to inhibit dHC or vHC glutamatergic neurons in a temporally precise manner before, during, or after the consumption of a meal, which consisted of either sucrose, standard chow, or sacharin. This allowed us to determine when these neurons are critical for limiting future intake and to identify whether neural inhibition restricted to the period following the consumption of a meal, when the memory of the meal would be undergoing consolidation, would increase subsequent intake at a later time when the neurons were no longer inhibited. Given that both dHC and vHC are implicated in memory consolidation (Oliveira et al., 2010; Holahan and Routtenberg, 2011; Zhu et al., 2014), we predicted that postmeal inhibition of either dHC or vHC would hasten meal initiation and increase future intake.

## Materials and Methods

### Subjects

Adult male Sprague Dawley rats (*N* = 94; postnatal day 52–58 on arrival; Charles River Laboratories) were single-housed in Optirat cages (Animal Care Systems). Unless otherwise stated, the rats were kept on a 12/12 h light/dark cycle and given *ad libitum* access to pelleted food and water in their home cages. All procedures were performed in compliance with the NIH guidelines for care of laboratory animals and approved by the Georgia State University Institutional Animal Care and Use Committee.

### Viral vectors

Recombinant serotype 5 adeno-associated virus (rAAV5) vectors containing CaMKIIα-eArchT3.0-eYFP or the control CaMKIIα-GFP (University of North Carolina Vector Core) were stored in aliquots (–80°C) until surgery. In the hippocampus, the CaMKIIα promoter limits expression to glutamatergic pyramidal cells (Sik et al., 1998; Stark et al., 2013, 2014; Butler et al., 2016). Illumination of transduced neurons activates the hyperpolarizing outward proton pump eArchT3.0, producing strong neural inhibition (Chow et al., 2010; Deisseroth, 2011; Yizhar et al., 2011; Huff et al., 2013).

#### Stereotaxic surgery

At least 1 week after arrival, the rats were anesthetized with 5% isoflurane (Henry Schein Impromed) in 1000 ml/min of oxygen (Airgas) and given penicillin (1500 IU, i.m.; Henry Schein Impromed) and carprofen (5 mg/kg, s.c.; Henry Schein Impromed). Anesthesia was maintained with 1–3% isoflurane gas mixed in 500 ml/min oxygen for the duration of the surgery. A 33-gauge injection needle was used to deliver the rAAV5 (0.5 µl) into dHC (AP: –3.7 mm, ML: +2.8 mm: DV: –4.0 mm from skull surface; Paxinos and Watson, 2007) of one hemisphere and vHC of the other (AP: –5.3 mm, ML: +5.1 mm, DV: –7.4 mm). These coordinates were selected based on previous research demonstrating that manipulations of these areas within the hippocampus impact memory processes (Bast et al., 2001; Oliveira et al., 2010; Czerniawski et al., 2011; Holahan and Routtenberg, 2011; Chia and Otto, 2013; Zhang et al., 2014). The hemispheres were counterbalanced across rats and the virus containing the same construct was injected in both hemispheres. The injection needle was left in place for 5 min after the end of the infusion to facilitate diffusion and the rats were given sterile saline (0.9%, 3.00 ml, s.c.; Hospira) at the end of surgery.

For rats used in the behavioral experiments, a second surgical procedure was performed at least 2 weeks later to implant fiber optic probes at each injection site. The probes were constructed using previously described procedures (Sparta et al., 2011; Huff et al., 2016). Briefly, a fiber optic (200 μm core; ThorLabs) was glued into a stainless-steel fiber ferrule assembly (Precision Fiber Products), and the ferrules were affixed to the head using surgical screws and dental acrylic. Plastic dust caps (Precision Fiber Products) were placed on each ferrule to protect the fiber optic core. Rats were given at least 1 week of recovery before behavioral testing.

### Slice preparation and electrophysiology

Patch-clamp electrophysiology recordings in acute dHC or vHC brain slice preparations were used to confirm the ability of eArchT3.0 to reliably and reversibly inhibit neuronal firing. Three to 4 weeks after the eArchT3.0 injections, rats (*n* = 5) were anesthetized with ketamine/xylazine (93/7 mg/kg, i.p., Henry Schein) and transcardially perfused with ice-cold, carbogen (95% O_2_/5% CO_2_, AirGas)-saturated cutting solution. The brain was then removed and 300-µm coronal (dHC) or horizontal (vHC) brain sections were cut in carbogen-saturated ice cold cutting solution using a vibrating-blade microtome. After sectioning, the brain slices were transferred to carbogen-saturated aCSF and incubated at ∼35°C for 30 min. The sections were kept at room temperature until they were transferred to the perfusion chamber for recording. The sucrose cutting solution contained: 205 mM sucrose, 2.5 mM KCl, 1.25 mM NaH_2_PO_4_, 7.5 mM MgCl_2_, 0.5 mM CaCl_2_, 11.1 mM glucose, and 21.4 mM NaHCO_3_. The aCSF contained: 126 mM NaCl, 2.5 mM KCl, 2.4 mM CaCl_2_, 1.2 mM NaH_2_PO_4_, 1.2 mM MgCl_2_, 11.1 mM glucose, and 21.4 mM NaHCO_3_. In a subset of experiments, kynurenic acid was included in the cutting solution (500–700 mM) and during the initial 35°C incubation in aCSF (1 µM).

For electrophysiology recording, a brain slice was transferred to the recording chamber and perfused constantly with room temperature, carbogen-saturated aCSF at a flow rate of 1.5–2.0 ml/min. Recordings were made using a potassium gluconate-based internal solution containing: 128 mM K gluconate, 10.0 mM HEPES, 10.0 mM NaCl, 1.0 mM MgCl_2_, 0.1 mM EGTA, 2.0 mM (Mg) ATP, 0.3 mM (Na) GTP, and 10.0 creatine phosphate. Electrodes had a resistance of 3.4–4.3 MΩ when filled with the potassium gluconate internal solution. Series resistance values ranged from 5–15 MΩ, and experiments were terminated and the cell excluded from analysis if the series resistance exceeded 20 MΩ. The data were sampled at 10 kHz and filtered at 2.6 kHz using an Axon MultiClamp 700B amplifier and Axograph X software. Neurons expressing eArchT3.0 were identified by eYFP fluorescence and were patch-clamped under gradient contrast optics. A ferrule fiber optic probe was connected to the laser and positioned above the slice at a ∼30–45° angle to activate eArchT3.0 during the electrophysiology recordings. After identification of a light-activated current in voltage clamp, neurons were recorded in the current-clamp configuration, and two approaches were used to test for the light-induced inhibition of neuronal firing (Kheirbek et al., 2013). (1) Neurons (*n* = 11) were given a depolarizing current injection for 4 s and green light (556 nm) was applied to the slice during the middle 2 s. The firing rate during the depolarizing current injection was measured before, during, and after light application, and the rates were normalized to the firing rate before light activation to allow for comparison between cells. (2) Brief, depolarizing current injections (50 ms) were applied to the neurons (*n* = 4) at 1 Hz to reliably elicit an action potential. After stable action potential generation was achieved for a minimum of 2.5 min, green light (556 nm) was continuously applied for 10 min (i.e., the duration used for the behavioral experiments), followed by additional monitoring for at least 2.5 min after the end of the light application. Action potential fidelity was calculated by determining the % of current injections eliciting an action potential at baseline, during light application, and in the post-light period. Cells were only included in the analyses if the recording remained stable during the entire experiment. Cells that became unstable or died during the course of the recording were not included in the analyses.

### Optical inhibition during behavior

Rats were connected to the laser by attaching their ferrules to a fiber optic leash using a Quick-Release interconnector (ADAF2; ThorLabs). The leash was attached to an optical commutator (RJPFF2; ThorLabs) allowing free rotation of the optic leashes. A FC/PC fiber coupler (Opto Engine LLC) connected the rotary joint to the laser source (200 mW DPSS laser, 556 nm; Opto Engine LLC). Light output was adjusted to allow for 10 mW from the fiber tip (Gradinaru et al., 2009; Yizhar et al., 2011; Huff et al., 2013, 2016) and was measured using an optical power meter (PM20A; ThorLabs). Ten mW light output produces ∼1 mW/mm^2^ of light up to 1 mm from the fiber tip and illumination (556 nm) activates eArchT3.0 in at least 0.4 mm^3^ of tissue (Yizhar et al., 2011).

### Sucrose consumption

The effects of optical inhibition of dHC or vHC glutamatergic neurons on intake of 32% (w/v) sucrose solution were tested. This sucrose solution was used as the meal because (1) it is very palatable/rewarding to rats (Hajnal et al., 2004; Smith, 2004), (2) its stimulus qualities are more specific than meals that include fats and proteins, and (3) it cannot be hoarded. To rule out any effects of novelty and to ensure that rats reliably consumed sucrose on presentation, the rats (eArchT3.0: *n* = 20; illumination-alone/no opsin control: *n* = 11) were exposed to the sucrose solution for 5 d before the optical manipulations. On the first exposure day, the rats were brought to the testing room at the beginning of the light cycle, placed into polycarbonate testing cages (22 × 43 × 22 cm) that did not contain any food, and then were given sucrose 8 h later for 10 min. The same procedure was repeated for the next 4 d, with the exception that sucrose was presented 3 h after they were placed into the testing cages. We started with an 8-h period without chow to increase the likelihood that the rats would approach the bottle, but then decreased it to 3 h to be within the range of an average postprandial intermeal interval (ppIMI; Snowdon, 1969).

On the experimental days, the rats were placed in the testing cages without food and then given sucrose 3 h later. They were connected to the laser 15 min before the sucrose was presented and were given sucrose for the duration of the 4-h experimental period. An experimenter measured latency to the first tube sipper tube contact using a Precision Solid State Time-It stopwatch (Petroleum Analyzer Company, L.P.). The testing cages were equipped with a modified lickometer system that measured the change in system resistance when a rat licked from a sipper tube (Model 86062, Lafayette Instruments). The Activity Wheel Monitoring Program (Lafayette Instruments) recorded all sipper tube contacts, which were operationally defined as any direct oral contact with the sipper tube longer than 3 s (Thaw et al., 1998). This criterion improved scoring reliability by virtually eliminating all sniffs as contacts. A meal was defined as any bout containing at least 30 licks (Smith, 2000; Hannapel et al., 2017), and this criterion was applied to the *post hoc* analyses of the licking measures. All sipper tube contacts were assumed to result in ingestion, and the amount consumed was estimated indirectly by summing the duration of all sipper tube contacts during the meal.

To identify when neural activity in dHC and vHC neurons is necessary for inhibiting intake, illumination was provided on the experimental days to dHC or vHC during one of three epochs: (1) for 10 min before the rats were given sucrose (before first meal condition); (2) during the first 10 min of the first sucrose meal (during first meal condition); or (3) for 10 min after the end of the first sucrose meal (after first meal condition). Intake was also assessed on another day in which the rats were attached to the laser, but not given illumination (none, non-illumination control condition). eArchT3.0 and control rats were given all seven treatment conditions (i.e., before, during, or after the first meal in dHC or vHC and none) in a counterbalanced order with at least 48 h separating each experimental day. This design allowed us to manipulate dHC and vHC in the same rat and decreased the number of animals needed by half. We found previously that unilateral dHC or vHC manipulations are sufficient to influence intake (Henderson et al., 2013; Hannapel et al., 2017).

To effectively time the manipulations given after the end of the first meal, it was critical to distinguish pauses within a meal from the end of a meal. Previous work indicates that when rats stop ingesting for 5 consecutive min there is a low likelihood that they will initiate eating again at that time (Zorrilla et al., 2005; Fekete et al., 2007) and a high probability that they will exhibit a progression of active grooming and resting behaviors known as the behavioral satiety sequence (Antin et al., 1975; Kushner and Mook, 1984; Zorrilla et al., 2005; Fekete et al., 2007). Based on this evidence, a meal was operationally defined as any period of consumption of at least 30 licks followed by 5 consecutive min without licking (Smith, 2000; Hannapel et al., 2017). The 30-lick criterion was applied *post hoc* to the analyses of the licking measures. One significant consequence of this operational definition is that 5 min had to elapse before the experimenter could know that the first meal was terminated and thus inhibition given after the first meal condition was actually started 5 min after the end of the first meal. Also, to restrict the inactivation to the postprandial period, the laser was turned off before 10 min if rats began to consume their second meal during the illumination. For inhibition given during the first meal, the laser was turned off if the rats stopped eating for 5 min during the illumination to minimize inhibition to ongoing intake.

To determine whether inactivation given before, during, or after the first meal accelerated the onset of the second meal and increased intake during the second meal, we measured the size of the first meal, the interval between the first and second meal (i.e., the ppIMI), and the size of the second meal. Rats that did not consume a second meal were given a ppIMI score of 4 h minus the latency to consume their first meal and the duration of that meal. Compared to smaller meals, larger meals are followed by a longer ppIMI, which is referred to as the postprandial correlation (Le Magnen and Tallon, 1963, 1966). Therefore, to control for differences in the size of the first meal between rats and between experimental days, we also used the satiety ratio (duration of ppIMI after first meal/size of the first meal) as a measure of the ppIMI.

### Chow consumption

The goal of this experiment was to determine if hippocampal neurons also limit future homeostatic feeding behavior by testing the effects of optical inhibition on chow intake at the beginning of the dark cycle when rats typically eat their first major chow meal (Clifton et al., 1984; Johnson and Johnson, 1990; Glendinning and Smith, 1994; Demaria-Pesce and Nicolaïdis, 1998; Clemens et al., 2014). Rats (eArchT3.0: *n* = 18; control: *n* = 8) in this experiment were placed on a reverse light-cycle schedule (12/12 h dark/light cycle) on arrival from the vendor and after 1 week of acclimation were given 3 d of habituation to the experimental procedures before behavioral testing. Specifically, the rats were moved to an illuminated testing room and placed in testing cages that did not contain chow 20 min before the end of the light phase. After 20 min, the room lights were turned off, a red light was turned on, and a glass Petri dish containing standard chow was placed into the cage for 2 h.

On experimental days, the rats were brought to the testing cages 20 min before the end of the light phase, connected to the laser, and then given chow 15 min later. Illumination was provided to dHC or vHC for 10 min either before chow was presented (before first meal condition), as soon as the rats started to ingest their first chow meal (during first meal condition), or after they stopped consuming their first chow meal (after first meal condition). A meal was operationally defined as any period of consumption of at least 0.25 g of chow followed by 5 consecutive minutes without ingestion (Azzara et al., 2002; Zorrilla et al., 2005; Kanoski et al., 2013; Hsu et al., 2015a, 2018). An experimenter blind to virus condition manually recorded the timing and amount of intake for 2 h after chow presentation, which entailed weighing the dishes after each 5 min pause in eating. The 0.25-g criterion was applied during the subsequent analyses of the eating measures; these *post hoc* analyses indicated that all rats consumed at least 0.25 g in all eating episodes that preceded 5-min breaks. Rats that did not consume a second meal were given a ppIMI score of 2 h minus the latency to consume their first meal and the duration of that meal.

### Saccharin intake

To test whether postmeal optical inhibition increased sucrose and chow intake by impairing processing of postprandial interoceptive visceral cues, a control experiment was conducted wherein we tested the effects of postmeal dHC and vHC glutamatergic inhibition on intake of a 0.2% (w/v) saccharin solution. Saccharin is a non-caloric sweetener that has minimal postingestive consequences (Mook et al., 1980; Renwick, 1985, 1986; Sclafani and Nissenbaum, 1985; Foletto et al., 2016). Importantly, in contrast to 32% sucrose and chow, whose intake is controlled by gastrointestinal and post-absorptive mechanisms (Strader and Woods, 2005; Cummings and Overduin, 2007), saccharin intake is controlled primarily by oral satiety (Hsiao and Tuntland, 1971; Mook et al., 1980, 1981; Kushner and Mook, 1984; Sclafani and Nissenbaum, 1985). As in the sucrose experiment, rats (eArchT3.0: *n* = 13) were given 5 d of pre-exposure to the saccharin solution, and then on the experimental days they were given either no illumination or dHC or vHC illumination for 10 min after they consumed their first saccharin meal (after first meal condition) in a counterbalanced order.

### Histology

After the completion of the behavioral experiments, the rats were euthanized with a lethal dose a pentobarbital (120 mg/kg; Henry Schein Impromed) and perfused transcardially with 4% paraformaldehyde (Fisher). Brains were harvested and left overnight in paraformaldehyde (4°C) and then transferred to a 30% sucrose and ethylene glycol antifreeze solution for at least 48 h. The brains were sectioned (50 µm) using a cryostat (CM3050 S; Lieca Biosystems) and mounted on gelatin-subbed slides using a Mowiol and DABCO antifade medium (Sigma Aldrich). dHC and vHC images were obtained using a fluorescent microscope (Axio Zoom V16; Zeiss), and viral expression and ferrule placement were visualized using Zeiss AxioVision imaging software (Carl Zeiss).

### Experimental design and statistical analysis

Different groups of rats were used for the electrophysiology, chow, sucrose, and saccharin consumption experiments. All statistical analyses and graphs were generated using IBM SPSS Statistics for Windows, version 21.0 (IBM Corporation), SigmaStat (v11.0, Systat Software, Inc.), and GraphPad Prism 7 for Windows (GraphPad Software). For the electrophysiology, the brief 2-s inhibition data were analyzed using a repeated-measures ANOVA on ranks and the 10-min inhibition exposure data were analyzed using a repeated-measures ANOVA with a Tukey’s *post hoc* test. For the behavioral experiments, only rats that had successful placements in both dHC and vHC and that underwent all optical inhibition treatment conditions were included in the analyses. Intake in rats given illumination of CaMKIIα-eArchT3.0-eYFP or the control CaMKIIα-GFP before, during, or after the first meal was compared to intake on a non-illumination day (i.e., none; within-subject design for each construct). The behavioral data were tested for normality and homogeneity of variance using Shapiro–Wilkes and Bartlett’s tests, respectively. The results of these tests indicated that sucrose and chow intake measures required non-parametric analyses. Consequently, Friedman tests and Dunn’s multiple comparisons *post hoc* tests were used to compare each optogenetic condition to the non-illumination condition. These data are represented in the figures as box-and-whiskers plots with median values placed in the center of each box, with the whiskers representing the minimum and maximum values. The saccharin data were normally distributed and had homogeneous variance and were thus analyzed with one-way repeated-measures ANOVAs with Geisser–Greenhouse correction and Bonferroni’s corrections for multiple comparisons. These data are also represented in the figures as box-and-whiskers plots to facilitate comparison with the sucrose and chow intake data.

## Results

### Activation of eArchT3.0 in dHC or vHC inhibited neuronal firing in a temporally-specific, steady, and reversible manner

**Figure 1.**
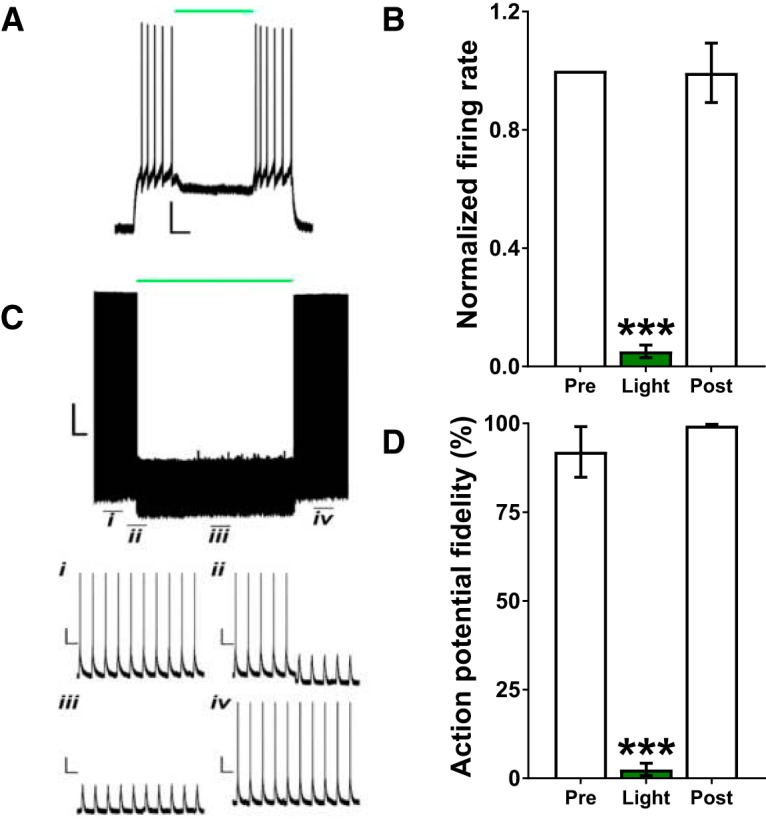
Optical stimulation of eArchT3.0 produced steady, temporally-specific, and reversible inhibition of dHC and vHC glutamatergic neurons. ***A***, Sample light-induced inhibition of a vHC-eArchT3.0-expressing neuron for 2 s (green line). Scale bar: 20 mV/0.5 s. ***B***, Light application for 2 s significantly decreased the mean (±SEM) firing rate of dHC and vHC-eArchT3.0-expressing neurons during that 2-s period (*n* = 11; dHC and vHC combined). ***C***, Sample light-induced inhibition of a vHC-eArchT3.0-expressing neuron for 10 min (green line); (**C*i***) at baseline, (**C*ii***) before, (**C*iii***) during, and (**C*iv***) after light. Scale bars: 20 mV/1 min (*i–iv*: 20 mV/1 s). ***D***, Light application for 10 min significantly decreased mean (±SEM) AP fidelity (*n* = 4; dHC and vHC combined); ^***^p < 0.0005 versus Pre (i.e., before light application) and Post (i.e., after light application was terminated).

Patch-clamp electrophysiology in acute slice preparations was used to test the ability of eArchT3.0 to reversibly inhibit neuronal activity in dHC and vHC (Kheirbek et al., 2013). In the first approach, a depolarizing current injection was given for 4 s, and 556 nm light was applied to the cell during the middle 2 s of the current injection. Cells from dHC (*n* = 6) and vHC (*n* = 5) were pooled and analyzed together because there were no differences between the effects of light on dHC versus vHC [cell location (i.e., dHC vs vHC): *F*_(1,18)_ = 0.1680, *p* = 0.6920; time: *F*_(2,18)_ = 72.6440, *p* = 0.0010, cell location × time: *F*_(2,18)_ = 0.0379, *p* = 0.9630]. Light application significantly decreased firing rate with a return to baseline activity following cessation of the light (*χ* ^2^(3) = 16.5450, *p* = 0.0009; Fig. 1*A*,*B*). In the second approach, brief depolarizing light pulses (50 ms) were applied to the cell at 1 Hz, and the cell was illuminated with constant 556 nm light for 10 min (i.e., the duration used in the behavioral experiments). dHC (*n* = 2) and vHC (*n* = 2) cells were pooled for this analysis because there were no apparent differences between the effects of illumination of dHC versus vHC cells. Continuous light significantly reduced the number of depolarizing steps that caused action potentials, with a return to baseline firing immediately on termination of the light (*F*_(2,6)_ = 137.0860, *p* = 0.0001; Fig. 1*C*,*D*).

### Histology

**Figure 2.**
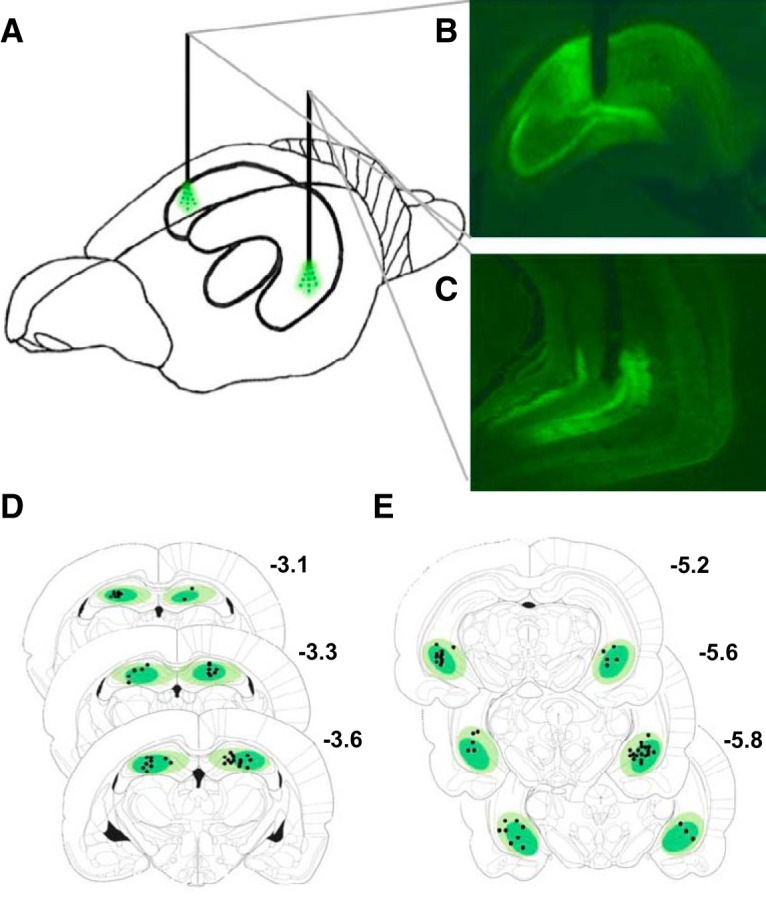
Depiction of virus expression and ferrule placement. ***A***, Depiction of ferrule location in dHC and vHC of the same rat. ***B***, Representative image of robust eArchT3.0-eYFP expression and ferrule location in dHC and in (***C***) vHC. ***D***, Schematic depiction of virus expression and ferrule placement relative to bregma in dHC and in (***E***) vHC. Atlas plates adapted from Paxinos and Watson (2007).

A total of 25 rats were excluded from analysis in the sucrose (*n* = 6 ArchT3.0; *n* = 4 control virus), chow (*n* = 6 ArchT3.0; *n* = 4 control virus), and saccharin (*n* = 5 ArchT3.0) experiments due to incorrectly located ferrules or opsin expression, insufficient opsin expression, or tissue damage in at least one hemisphere. Figure 2 provides a schematic depiction of the location of the ferrules in dHC and vHC of the same animal (Fig. 2*A*), photomicrographs depicting representative virus expression in dHC (Fig. 2*B*) and vHC (Fig. 2*C*), and schematic depictions of virus distribution and ferrule locations for dHC (Fig. 2*D*) and vHC (Fig. 2*E*).

### Optical inhibition of dHC or vHC glutamatergic neurons given during or after the first sucrose meal decreased the latency to the second sucrose meal. Only inhibition given after intake increased the amount consumed during the second meal when the neurons were no longer inhibited

**Figure 3.**
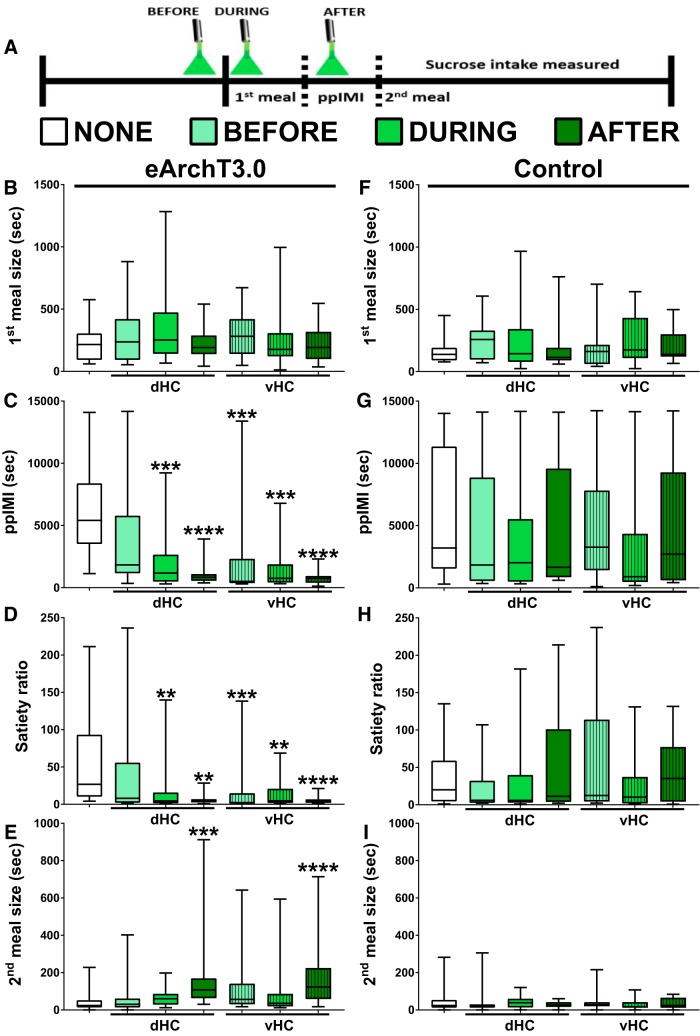
Postmeal inhibition of dHC or vHC glutamatergic neurons promoted sucrose meal initiation and increased future sucrose intake. ***A***, Timeline showing when optical inhibition of dHC or vHC glutamatergic neurons was given for 10 min relative to the first sucrose meal on different experimental days. All of the rats were given all of the seven treatment conditions (i.e., within-subject design) in a counterbalanced order. ***B***, Optical inhibition given before, during, or after the first sucrose meal did not affect the size of the first meal. Optical inhibition given during or after the first meal (***C***) decreased the ppIMI and (***D***) satiety ratio, whereas only inhibition after the first meal (***E***) increased the amount eaten during the second meal, even though the neurons were no longer inactivated during intake of that second meal (*n* = 20; within-subject). Inhibition of vHC glutamatergic neurons given before intake of the first meal decreased the ppIMI and satiety ratio, but did not affect the other measures. Illumination given before, during, or after the first sucrose chow meal did not affect (***F***) the size of the first meal (***G***) the ppIMI, (***H***) the satiety ratio, or (***I***) the size of the second meal in the no opsin control rats (*n* = 11; within-subject). The central line depicts the median and the whiskers represent the maximum-minimum data points for each condition; ^**^p < 0.005; ^***^p < 0.0005; ^****^p < 0.0001 versus none.

This experiment tested the effects of inhibition of dHC or vHC glutamatergic neurons given before, during, or after the first sucrose meal on the amount consumed during the first meal, the timing of the second meal (i.e., ppIMI and satiety ratio), and the size of the second meal (Fig. 3*A*). The goal was to identify when neural activity in these neurons is necessary for inhibiting intake and to determine whether neural inhibition given for 10 min after the first sucrose meal, when the memory of the meal would be undergoing consolidation, would promote the initiation of the next meal and increase intake during that next meal when the neurons were no longer inhibited. Friedman tests indicated that optical activation of eArchT3.0 in dHC and vHC did not affect the size of the first sucrose meal (*χ* ^2^(6) = 7.36, *p* = 0.2887; Fig. 3*B*) nor licking rates during the first meal (*χ* ^2^(6) = 7.07, *p* = 0.3142), but did significantly affect the duration of the ppIMI (*χ* ^2^(6) = 39.4, *p* = 0.0001; Fig. 3*C*), magnitude of the satiety ratio (*χ* ^2^(6) = 26.4, *p* = 0.0002; Fig. 3*D*), and the size of the second sucrose meal (*χ* ^2^(6) = 35.3, *p* = 0.0001; Fig. 3*E*).

Dunn’s *post hoc* tests indicated that the effects of optical inhibition on the ppIMI, satiety ratio, and size of the second meal depended on the timing and location of the inhibition. Specifically, compared to intake on a day in which illumination was not given (i.e., none), inhibition of vHC glutamatergic neurons given before the first sucrose meal accelerated the onset of the second sucrose meal (i.e., decreased the ppIMI: *p* = 0.0001 and satiety ratio: *p* = 0.0003; Fig. 3*C*,*D*). In contrast, dHC inhibition given before the first sucrose meal did not affect these measures (ppIMI: *p* = 0.4037; satiety ratio: *p* = 0.0770). Inhibition of either dHC or vHC given during or after intake of the first sucrose meal also decreased the ppIMI (during, dHC: *p* = 0.0011; vHC: *p* = 0.0003, after, dHC: *p* = 0.0001; vHC: *p* = 0.0001; Fig. 3*C*) and satiety ratio (during, dHC: *p* = 0.0027; vHC: *p* = 0.0035, after, dHC: *p* = 0.0020; vHC: *p* = 0.0001; Fig. 3*D*). Interestingly, only inhibition of dHC or vHC given after the first sucrose meal increased the amount consumed during the second meal (dHC: *p* = 0.0002; vHC: *p* = 0.0001; Fig. 3*E*); inhibition given before or during the first sucrose meal did not affect the size of the second meal (before, dHC: *p* = 0.9999; vHC: *p* = 0.1535, during, dHC: *p* = 0.5538; vHC: *p* = 0.9999).

These effects of optical inhibition on the timing and amount of sucrose consumed were not due to the order of the manipulations across the experimental days [first meal size (*χ* ^2^(6) = 4.53, *p* = 0.6059); ppIMI (*χ* ^2^(6) = 9.00, *p* = 0.1736); satiety ratio (*χ* ^2^(6) = 9.81 *p* = 0.1327); second meal size (*χ* ^2^(6) = 3.68, *p* = 0.0576)], and the increase in the size of the second meal was not due to increased licking rate during the consumption of that meal (*χ* ^2^(6) = 8.25, *p* = 0.2201). Importantly, the Friedman tests showed that illumination of the control virus did not affect sucrose meal size or meal timing [first meal size (*χ* ^2^(6) = 4.2, *p* = 0.6494; Fig. 3*F*); ppIMI (*χ* ^2^(6) = 3.7, *p* = 0.7175; Fig. 3*G*); satiety ratio (*χ* ^2^(6) = 5.16 *p* = 0.5233; Fig. 3*H*); second meal size (*χ* ^2^(6) = 1.82, *p* = 0.9356; Fig. 3*I*)].

### Postmeal optical inhibition of dHC or vHC glutamatergic neurons also increased future intake of standard chow

**Figure 4.**
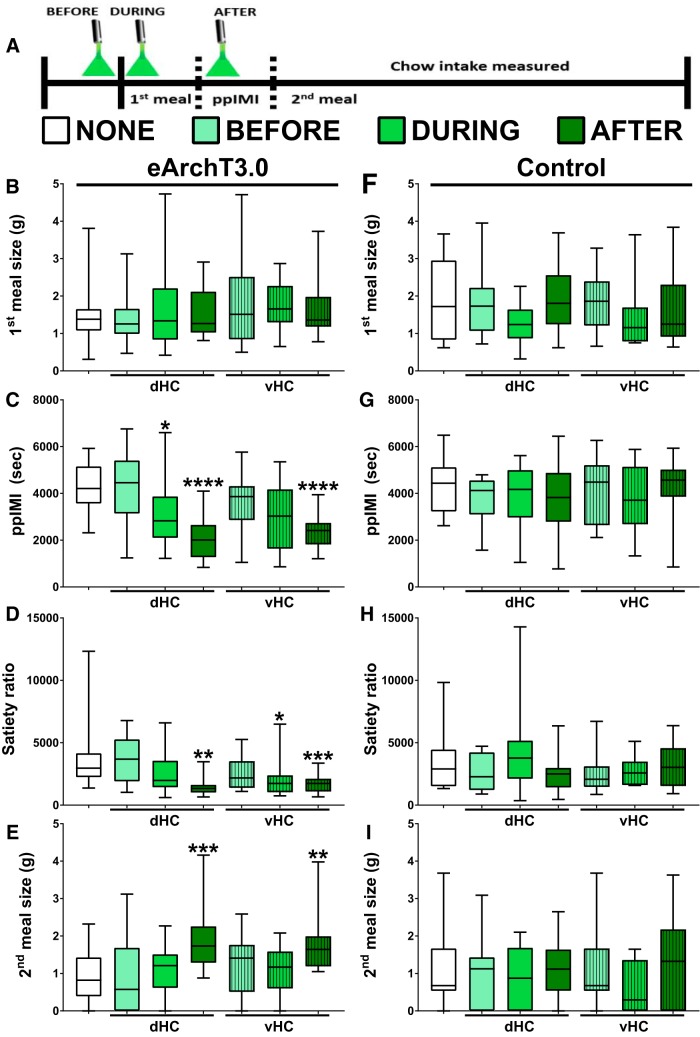
Postmeal inhibition of dHC or vHC glutamatergic neurons also promoted chow meal initiation and increased future chow intake. ***A***, Timeline showing when optical inhibition of dHC or vHC glutamatergic neurons was given for 10 min relative to the first chow meal on different experimental days. All of the rats were given all of the seven treatment conditions (i.e., within-subject design) in a counterbalanced order. Optical activation of eArchT3.0 given before intake of the first meal did not affect (***B***) the amount eaten during the first meal or any of the other measures, whereas optical inhibition given during or after the first meal (***C***) decreased the ppIMI and (***D***) satiety ratio. Only inhibition given after the first meal (***E***) increased the amount eaten during the second meal, even though the neurons were no longer inactivated during intake of that meal (*n* = 18; within-subject). Illumination given before, during, or after the first chow meal did not affect (***F***) the size of the first meal (***G***) the ppIMI, (***H***) the satiety ratio, or (***I***) the size of the second meal in the no opsin control rats (*n* = 8; within-subject). The central line depicts the median and the whiskers represent the maximum-minimum data points for each condition; ^*^p < 0.05; ^**^p < 0.005; ^***^p < 0.0005; ^****^p < 0.0001 versus none.

This experiment determined whether dHC and vHC glutamatergic neurons also limit future homeostatic feeding behavior. Inhibition of dHC or vHC glutamatergic neurons was given before, during, or after the first chow meal of the dark cycle (Fig. 4*A*). As in the case of sucrose, the results of the Friedman tests indicated that optical illumination of eArchT3.0 did not affect the size of the first chow meal (*χ* ^2^(6) = 8.17, *p* = 0.2261; Fig. 4*B*), but did affect the timing of the next chow meal [i.e., the ppIMI duration (*χ* ^2^(6) = 39.90, *p* = 0.0001; Fig. 4*C*) and satiety ratio (*χ* ^2^(6) = 32.00, *p* = 0.0001; Fig. 4*D*)] and the amount consumed during the second meal (*χ* ^2^(6) = 23.90, *p* = 0.0005; Fig. 4*E*).

Dunn’s *post hoc* tests indicated that the effects of inhibition were dependent on the timing of the inhibition relative to intake and on the anatomical location of the inhibition. dHC or vHC illumination given before intake of the first meal did not affect the timing of the second meal [ppIMI: dHC: *p* = 0.9999; vHC: *p* = 0.9999 (Fig. 4*C*), satiety ratio: dHC: *p* = 0.9999; vHC: *p* = 0.9999 (Fig. 4*D*)] or the amount consumed during the second meal (dHC: *p* = 0.9999; vHC: *p* = 0.1371; Fig. 4*E*). Illumination given during the first meal only affected the timing of the next meal but not the amount consumed during that meal. Specifically, dHC inhibition given during the first chow meal significantly decreased the ppIMI (*p* = 0.0329; Fig. 4*C*), with a similar trend for vHC inhibition given during the first meal (*p* = 0.0654; Fig. 4*C*). vHC inhibition given during the first meal but not dHC inhibition given at that time decreased the satiety ratio (dHC: *p* = 0.7369; vHC: *p* = 0.0329; Fig. 4*D*). Neither dHC nor vHC inhibition given during the first meal affected the size of the second meal (during, dHC: *p* = 0.9999; vHC: *p* = 0.7369; Fig. 4*E*). As in the case of sucrose, only illumination given after the first meal promoted meal initiation and increased intake during that second meal. Optical inhibition of dHC or vHC given after the first chow meal significantly accelerated the onset of the next meal [i.e., decreased the ppIMI duration, dHC: *p* = 0.0001; vHC: *p* = 0.0001 (Fig. 4*C*) and the satiety ratio, dHC: *p* = 0.0023; vHC: *p* = 0.0009 (Fig. 4*D*)] and increased the amount consumed during the second meal (dHC: *p* = 0.0007; vHC: *p* = 0.0094; Fig. 4*E*).

As in the case of sucrose, these effects of optical inhibition on the timing and amount of chow consumed were not due to the order of the manipulations across experimental days [first meal size (*χ* ^2^(6) = 3.45, *p* = 0.9022); ppIMI (*χ* ^2^(6) = 8.07, *p* = 0.2329); satiety ratio (*χ* ^2^(6) = 8.48 *p* = 0.2052); second meal size (*χ* ^2^(6) = 3.02, *p* = 0.8058)]. Of note, optical illumination of the control virus did not affect any of the intake measures [first meal size (*χ* ^2^(6) = 5.17, *p* = 0.5225; Fig. 4*F*); ppIMI (*χ* ^2^(6) = 2.04, *p* = 0.9164; Fig. 4*G*); satiety ratio (*χ* ^2^(6) = 8.2 *p* = 0.2241; Fig. 4*H*); second meal size (*χ* ^2^(6) = 6.49, *p* = 0.3707; Fig. 4*I*)].

### Postmeal optical inhibition of dHC or vHC glutamatergic neurons increased future intake of the noncaloric sweetener saccharin

**Figure 5.**
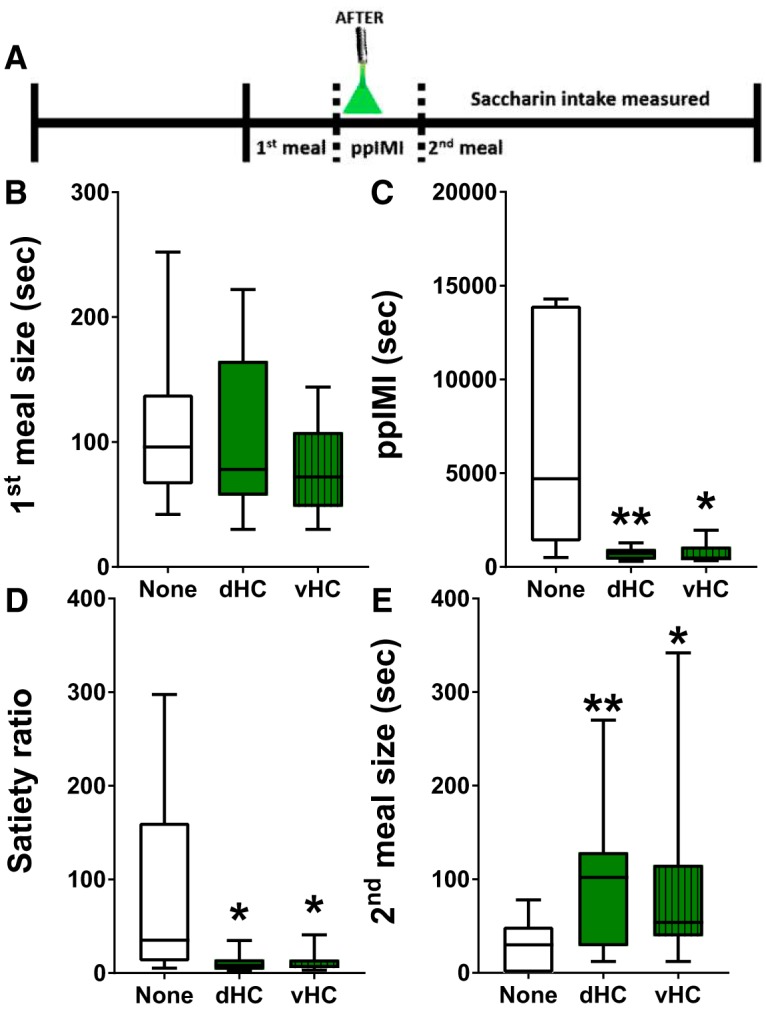
Postmeal inhibition of dHC or vHC glutamatergic neurons promoted future intake of the noncaloric sweetener saccharin. ***A***, Timeline showing that optical inhibition of dHC or vHC glutamatergic neurons was given for 10 min after intake of the first saccharin meal. All of the rats (*n* = 13) were given all three treatment conditions (i.e., none and after in dHC or vHC; within-subject design) in a counterbalanced order. Inhibition given after the first saccharin meal (***B***) did not affect the amount consumed during the preinhibition meal but did (***C***) decrease the ppIMI and (***D***) satiety ratio and (***E***) increase the amount consumed during the second saccharin meal, even though these neurons were not inhibited during intake of that second meal. The central line depicts the median and the whiskers represent the maximum-minimum data points for each condition; ^*^p < 0.05; ^**^p < 0.005 versus none.

It is possible that postmeal manipulations promoted meal initiation and increased subsequent intake by disrupting hippocampal processing of postprandial interoceptive visceral signals (Davidson et al., 2014; Hsu et al., 2015a; Stevenson and Francis, 2017). To address this, this experiment determined whether postmeal optogenetic inhibition of dHC or vHC glutamatergic neurons would promote future intake of saccharin, which produces minimal postingestive consequences (Mook et al., 1980; Renwick, 1985, 1986; Sclafani and Nissenbaum, 1985; Foletto et al., 2016). The results indicated that activation of eArchT3.0 in dHC or vHC glutamatergic neurons given after the first saccharin meal decreased the ppIMI (*F*_(1.01,12.20)_ = 16.30, *p* = 0.0016; Fig. 5*C*) and satiety ratio (*F*_(1.03,12.30)_ = 7.65, *p* = 0.0163; Fig. 5*D*) and increased the size of the second saccharin meal (*F*_(1.57,20.40)_ = 5.05, *p* = 0.0226; Fig. 5*E*). Bonferroni *post hoc* analyses showed that this effect was produced by inhibition of either dHC or vHC [ppIMI, dHC: *p* = 0.0026; vHC: *p* = 0.0040 (Fig. 5*C*); satiety ratio, dHC: *p* = 0.0270; vHC: *p* = 0.0270 (Fig. 5*D*); second meal size, dHC: *p* = 0.0035; vHC: *p* = 0.0492 (Fig. 5*E*)]. As expected, the size of the first meal, which was consumed immediately before the inhibition, did not differ between the treatment conditions (*F*_(1.76,21.20)_ = 1.23, *p* = 0.3090).

## Discussion

The present results indicate that neural activity in principal dHC and vHC neurons is necessary during the early postprandial period for limiting future intake. Inhibition of dHC or vHC glutamatergic neurons given after the end of a meal increased the size of the subsequent meal when the neurons were no longer inhibited. The results also suggest that, although these neurons inhibit future intake, they do not limit current intake as optical inhibition given during a meal did not affect the amount consumed during that meal. Electrophysiological recordings showed that neural activity returned to baseline immediately on termination of the 10 min of inhibition, supporting the inference that neural activity was not inhibited during intake of the second meal. Optogenetic inhibition increased future sucrose and chow consumption, indicating that dHC and vHC principal neurons inhibit future homeostatic and hedonic feeding behavior. The finding that postmeal optogenetic inhibition increased future saccharin intake suggests that the ability of dHC and vHC glutamatergic neurons to control future intake does not require postprandial interoceptive visceral signals because saccharin ingestion produces minimal postingestive consequences and saccharin meal timing and size are determined primarily by oropharyngeal processes (Kushner and Mook, 1984; Renwick, 1985, 1986; Sclafani and Nissenbaum, 1985). It would be interesting to test whether hippocampal inhibition would have the opposite effects on saccharin intake in rats given long-term exposure to saccharin because chronic saccharin intake can impair the learned relationship between sweet orosensation and caloric load and lead to subsequent overconsumption (Davidson et al., 2011; Swithers, 2015).

The present results also show that inhibition given during or after the consumption of a meal accelerated the onset of the next meal, suggesting that neural activity at different time points relative to ingestion influence meal initiation (i.e., during and after intake) versus future meal size (i.e., only after intake). The finding that inhibition given during a meal did not produce as robust an effect on future intake as did inhibition given after a meal also suggests that hippocampal neurons receive most of the neural signals necessary for inhibiting future intake during the postprandial period. The fact that postmeal inhibition did not commence until 5 min after the termination of the first meal (see methods) suggests that hippocampal neural activity required to control future intake persists for >5 min after the meal.

Optical inhibition given for 10 min on multiple days did not appear to produce any long-term dysfunction. Our electrophysiological recordings showed that neural activity returned to baseline when the 10 min-illumination was terminated, consistent with the finding that 15 min of continuous eArchT3.0 illumination inhibits neural activity without producing desensitization or cellular damage (Huff et al., 2013; Stefanik et al., 2013; Tsunematsu et al., 2013; Takahashi et al., 2014). Our behavioral findings showed that the effects of inhibition are specific to certain times and measures although the rats were given inhibition at all time points in a counterbalanced order, and we confirmed that intake did not change across experimental days. Importantly, our illumination protocol did not increase intake of sucrose or chow in rats given the control vector.

Our previous results showing that dHC and vHC muscimol infusions given after a sucrose meal accelerated the onset of the next sucrose meal and increased sucrose intake (Henderson et al., 2013; Hannapel et al., 2017) did not distinguish between different periods relative to the meal and could have been due to the effects of the muscimol persisting through the next meal (Martin, 1991; Arikan et al., 2002). Moreover, the findings did not specifically implicate principal neurons because muscimol could have inhibited several hippocampal neuronal types that express GABA_A_ receptors (Semyanov et al., 2003; Glykys et al., 2007; Mann and Mody, 2010). Thus, the use of temporally and genetically targeted manipulations in the present study significantly advances our understanding of hippocampal control of intake by providing compelling evidence that post-meal activity in principal hippocampal neurons is critical for limiting future intake.

Several lines of evidence suggest that postmeal hippocampal inhibition likely increased future intake via effects on the consolidation of the memory of the first meal and that principal dHC and vHC neurons are a critical component of the neural mechanisms that underlie the ability of meal-related memory to inhibit future intake in human participants (Robinson et al., 2012, 2013a,b, 2014). First, dHC or vHC manipulations given immediately after training in memory tasks impair subsequent retention (Cimadevilla et al., 2008; Oliveira et al., 2010; Holahan and Routtenberg, 2011; Fantin et al., 2013; Zhu et al., 2014), indicating that neural activity in dHC and vHC neurons during the period following an event is critical for memory consolidation. Second, dHC and vHC are necessary for many aspects of food/meal-related memories, such as food location, when food is available and food reward; (McDonald and White, 1993; Chinnakkaruppan et al., 2014; Kanoski and Grill, 2015). Third, our previous research found that ingestion of sucrose or saccharin activates molecular processes critical for synaptic plasticity and memory formation in dHC and vHC during the postprandial period (Henderson et al., 2016; Hannapel et al., 2017; Ross et al., 2019). For instance, consistent with studies examining other types of learning (Guzowski et al., 2001; Vazdarjanova et al., 2006; Czerniawski et al., 2011; Chia and Otto, 2013), our previous work found that sucrose and saccharin ingestion increased hippocampal expression of activity-regulated cytoskeletal-associated protein Arc mRNA (Henderson et al., 2016; Hannapel et al., 2017), which is necessary for memory consolidation (Guzowski et al., 2000). Fourth, the present finding that hippocampal inhibition given during a meal did not affect the amount consumed during that meal but did affect future intake is consistent with results from human participants showing that manipulating hippocampal-dependent memory encoding while eating has a bigger effect on intake at the next eating episode than on intake of the meal being remembered (Robinson et al., 2013b). Finally, our findings showing that inhibition given before intake does not affect subsequent eating behavior and that inhibition given during intake does not affect the amount eaten during that bout argues strongly against the possibility that inhibition impairs retrieval or use of information provided by memories of previous meals and/or the processing of preprandial physiologic signals. Thus, postmeal inhibition likely increased future intake by impairing memory consolidation and our results suggest that sweet orosensation is sufficient for hippocampal neurons to form and consolidate a meal-related memory and limit future intake and that post-ingestive interoceptive signals are not necessary. It is possible that impairing the consolidation of a memory of a meal accelerates meal onset and increases meal size by interfering with the ability of the meal-related memory to signal that an otherwise expected appetitive outcome will not be forthcoming (Jones et al., 2018).

Synaptic plasticity at hippocampal excitatory synapses is a critical mechanism underlying memory formation (Bailey et al., 2015). Increased synaptic strength in hippocampus augments functional connectivity between hippocampus and other brain regions (Canals et al., 2009), thereby providing a potential mechanism for hippocampal inhibition of intake. dHC neurons may modulate intake via longitudinal projections to vHC (Tamamaki et al., 1984, 1988; Amaral and Witter, 1989; Ishizuka et al., 1990; Yang et al., 2014), which is the source of most hippocampal projections to brain areas involved in eating (Kanoski and Grill, 2015). In support, activation of vHC glutamatergic projections inhibits feeding behavior (Sweeney and Yang, 2015; Hsu et al., 2018). Of note, activation of dHC Glu neurons increases neural activity in vHC, but not vice versa (Takata et al., 2015). This could account for similar effects of dHC and vHC inhibition and for finding that vHC inhibition produced more effects than dHC inhibition. Given their roles in memory consolidation, it would be expected that inhibition of dHC or vHC given during the postprandial period would have a similar effect on future intake. This does not mean, though, that both hippocampal sub-regions serve identical roles in regulating feeding behavior. For instance, unlike dHC, vHC is also implicated in motivational aspects of feeding and is more sensitive to food-related hormonal signals than dHC (Kanoski et al., 2011, 2013; Fitzpatrick et al., 2016).

The present results showed that hippocampal neural activity during and after ingestion is critical for influencing meal timing. Compared to our knowledge of the neural controls of meal size, there is a large gap in our understanding of how the brain inhibits meal initiation and influences the duration of the ppIMI. The ppIMI determines meal frequency and thus also affects total intake, an important issue because increased meal frequency (i.e., snacking) coincides with the increased prevalence of diet-induced obesity (Nielsen et al., 2002; Cutler et al., 2003; Nicklas et al., 2003). vHC inhibition given before intake of a scheduled sucrose meal also accelerated the onset of the next meal but did not affect the timing of spontaneous chow meals, raising the possibility that neural activity in vHC but not dHC glutamatergic neurons during the anticipation of a highly palatable meal influences the future timing of these meals.

The current results also provide a mechanism by which hippocampal dysfunction and obesity produce a positive feedback loop that leads to more hippocampal pathology and weight gain. Excess intake of fats and/or sugars and obesity in rodents impair hippocampal synaptic plasticity (Stranahan et al., 2008; Grillo et al., 2011; Karimi et al., 2013) and hippocampal-dependent memory (Pathan et al., 2008; Ross et al., 2009, 2012; Darling et al., 2013; Beilharz et al., 2014; Erion et al., 2014). Hippocampal dysfunction, in turn, increases meal frequency and food intake (Osborne and Dodek, 1986; Davidson and Jarrard, 1993; Clifton et al., 1998; Henderson et al., 2013; Hannapel et al., 2017) and promotes weight gain (Davidson et al., 2009, 2010; Sample et al., 2016). In humans, being overweight or obese is associated with hippocampal atrophy (Shefer et al., 2013; Bauer et al., 2014; Cherbuin et al., 2015) and episodic memory deficits (Cheke et al., 2016), and enhancing the memory of a meal may be a promising strategy for limiting intake and promoting weight loss (Robinson et al., 2013a,b, 2014).

## Synthesis

Reviewing Editor: Mark Baxter, Mount Sinai School of Medicine Decisions are customarily a result of the Reviewing Editor and the peer reviewers coming together and discussing their recommendations until a consensus is reached. When revisions are invited, a fact-based synthesis statement explaining their decision and outlining what is needed to prepare a revision will be listed below. Note: If this manuscript was transferred from JNeurosci and a decision was made to accept the manuscript without peer review, a brief statement to this effect will instead be what is listed below.

The manuscript was accepted after editorial review.
